# Atmospheric Pressure Plasma Chemical Vapor Deposition of Carvacrol Thin Films on Stainless Steel to Reduce the Formation of *E. Coli* and *S. Aureus* Biofilms

**DOI:** 10.3390/ma13143166

**Published:** 2020-07-15

**Authors:** Tsegaye Gashaw Getnet, Gabriela F. da Silva, Iolanda S. Duarte, Milton E. Kayama, Elidiane C. Rangel, Nilson C. Cruz

**Affiliations:** 1 Laboratory of Technological Plasmas, São Paulo State University, Sorocaba 18087-180, SP, Brazil; tsegshchem2004@gmail.com (T.G.G.); elidiane.rangel@unesp.br (E.C.R.); 2 Department of Chemistry, Bahir Dar University, Bahir Dar 79, Ethiopia; 3 Laboratory of Environmental Microbiology, Federal University of Sao Carlos, Sorocaba 18052-780, SP, Brazil; gfiori.silva@gmail.com (G.F.d.S.); iolanda.duarte@gmail.com (I.S.D.); 4 Laboratory of Plasmas and Applications, São Paulo State University, Guaratinguetá 12516-410, SP, Brazil; ekayama@gmail.com or

**Keywords:** dielectric barrier discharge, carvacrol plasma polymerization, biofilm inhibition

## Abstract

In this paper, we have investigated the deposition of thin films from natural carvacrol extract using dielectric barrier discharge (DBD) plasma polymerization, aiming at the inhibition of bacteria adhesion and proliferation. The films deposited on stainless steel samples have been characterized by scanning electron microscopy, infrared reflectance-absorbance spectroscopy, profilometry, and contact angle measurements. Films with thicknesses ranging from 1.5 μm to 3.5 μm presented a chemical structure similar to that of carvacrol. While the formation of biofilm was observed on untreated samples, the coating completely inhibited the adhesion of *E. coli* and reduced the adhesion of *S. aureus* biofilm in more than 90%.

## 1. Introduction

The excellent physical and chemical properties of metals make them very suitable for biomedical applications in medical devices, implants, and tissue engineering. Particularly in underdevelopment countries, stainless steel is widely used as implants and prosthesis in orthopedic surgeries for being cheaper than titanium and other more inert alloys. In spite of the relatively good clinical results, the bacterial colonization on such material is still a subject demanding attention. As is well known, many bacteria are able to attach and form complex colonies, known as biofilms, on various materials, including stainless steel [1]. Once established, the eradication of biofilms is quite difficult as they present resistance to antibiotics that can be up to 10^3^ times higher than that of planktonic bacteria [2,3,4]. Frequently, the only solution to extirpate the bacterial colonization is to surgically remove the infected implant, which, in addition to high economic costs, results in extra risks of morbidity and mortality to the patients. Thereby, methods of controlling harmful pathogenic biofilms are urgently required. In this regard, antimicrobial active coatings on biomaterials have gained significant interest over the past decade [5,6,7,8,9]. However, potential health and safety risks associated with the release of synthetic antimicrobials could limit the application of such materials [9]. In this context, the well-known hazardous effects of most synthetic biocidal compounds and the need to develop cheap and eco-friendly coating processes have stimulated studies focusing on the use of natural bacterial inhibitors. Numerous studies have highlighted the immense potential of natural compounds as antimicrobial products in food packaging, pharmaceuticals, and hygiene [10,11,12]. Furthermore, natural products have been considered as promising precursors for plasma polymerization. As an illustration, coatings produced from plasmas containing essential oils such as terpineol, linalyl acetate, and γ-terpinene have been demonstrated to be useful in different applications such as insulating and encapsulation layers in organic electronics, and biocompatible coatings for implants [13,14].

Several studies [15,16,17,18,19] have demonstrated the biocidal activity of carvacrol, a natural oil extracted from oregano, against free-standing micro-organisms. In addition, its use has been approved by the European Parliament and Council and by the U.S. Food and Drug Administration [20,21,22]. However, its activity, especially when immobilized on a surface, in inhibiting biofilm formation is still a subject under investigation. In order to fill this gap, in this work, to the best of our knowledge, for the first time, the feasibility of the deposition of carvacrol thin films by dielectric barrier discharge (DBD) plasmas to avoid biofilm formation on stainless steel samples has been evaluated.

## 2. Materials and Methods 

### 2.1. Sample Preparation 

Prior to depositions, 1 cm × 1 cm stainless steel samples were mirror polished and then cleaned in ultrasonic baths using detergent, distilled water, and isopropyl alcohol. Subsequently, the samples were dried with a hot air gun and stored in Petri dishes until use.

### 2.2. Plasma Polymerization of Essential Oil

**Figure 1:**
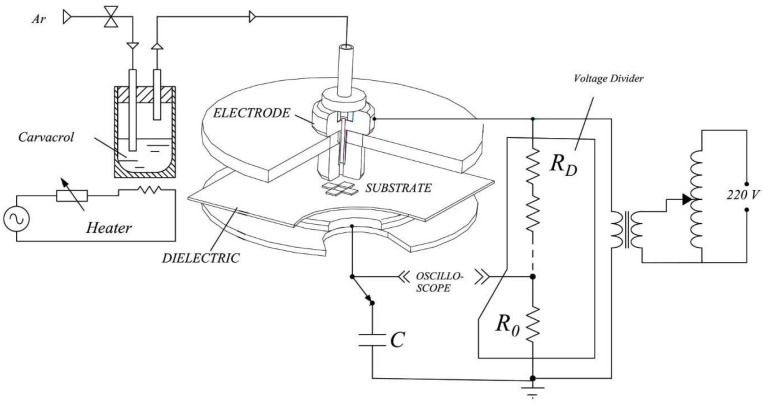
Schematic representation of the experimental setup.

Film deposition was performed in an especially designed dielectric barrier discharge reactor depicted in Figure 1. It consists of two parallel circular brass electrodes, 3 mm apart from each other, fitted axially in two parallel Polytetrafluoroethylene PTFE discs. A polyester sheet on the grounded lower electrode served as a dielectric barrier, while sinusoidal AC pulses (60 Hz, 15 kV maximum amplitude) were applied to the upper electrode. Carvacrol (5-Isopropyl-2-methylphenol, (CH_3_)_2_CHC_6_H_3_(CH_3_)OH), at least 98% pure, was kept in a temperature controlled-stainless steel bottle. Argon, at a flow of 5 L/min, was used as a monomer carrier gas as well as to help in plasma ignition. A 1000× voltage divider and a Tektronix TDS1001C, Tektronix China Co. Ltd., Shanghai, China, (30 MHz, 500 MS/s) oscilloscope were used to measure the applied voltage *v_A_* and the voltage drop *vc* through a 10 nF-capacitor. Such values were recorded and used to calculate the plasma excitation power as follows:

(1) $$P=\;\frac{1}{T}\;\int_{0}^{Q}v_{A}dq$$

where *T* is the period of the voltage pulse, *Q* is the charge stored in the capacitor during one period, and *q* is the instantaneous charge. According to such calculation, the average power delivered during the depositions was 0.54 ± 0.04 W.

The deposition time ranged from 15 to 45 min and the chemical structure and stability of the films were characterized by infrared reflectance absorbance spectroscopy (IRRAS), using a model FT/IR-410 Fourier-transform infrared spectrometer (Jasco Corp., Tokyo, Japan), co-adding 128 scans with a resolution of 4 cm^−1^. The surface morphology of the samples was also inspected by scanning electron microscopy (SEM) (JSM-6010LA, JEOL Ltd., Peabody, MA, USA), at 3 kV acceleration voltage and beam diameter and working distance of 3.5 nm and 10 mm, respectively. X-ray energy dispersive spectroscopy (EDS) was performed with a Dry SD Hyper EX-94410T1L11 detector (JEOL Ltd., Peabody, MA, USA), coupled to the SEM microscope, to characterize the chemical composition of the films. The EDS spectra were acquired with 5 and 10 kV electron beam energy with a beam diameter and a working distance of 6 nm and 10 mm, respectively. To avoid charging effects during the SEM inspections, samples were coated with a thin conducting layer of a golden-palladium. The roughness of the films was determined with a surface profilometer model Veeco D150 (Veeco Metrology, Tucson, AZ, USA). To evaluate the thickness, the films were deposited on stainless steel slides partially masked with Kapton tape and the height of the step formed after removing the tape was also measured with the profilometer. Thickness and roughness were measured at least 10 times in different locations on each sample. Wettability and surface energy of the films were evaluated from contact angle measurements with deionized water and diiodomethane as probe liquids in a goniometer Ramé-Hart 100-00 (Ramè-hart Instrument Co., Succasunna, NJ, USA). Surface energy was calculated using the harmonic mean method proposed by Owens-Wendt [23], which has been considered the most universal procedure for such evaluation.

### 2.3. Biofilm Assay

Escherichia coli (ATCC®11229, Campinas, SP, Brazil) and Staphylococcus aureus (ATCC® 6538) strains were used for biofilm assay. Initially, each stock strain was spread on tryptic soy agar (TSA) media, separately and incubated at 37 °C for 48 h [24,25]. Three samples produced with each treatment time were introduced separately in test tubes containing 5 mL of tryptone soy broth (TSB) with standardized inoculum concentration of 3.6–5 × 10^8^ colony forming unit (CFU)/mL. Then, the tubes were incubated for 3 h in a static oven at 36.5 °C for the adhesion phase. After this phase, the slides were withdrawn and washed two times with 3 mL of saline solution (0.85%) and transferred to sterile test tubes containing 5 mL of TSB culture medium, followed by incubation in a static oven at 36.5 °C for 24 h for biofilm formation. Afterward, the samples were transferred to a new test tube containing 10 mL of saline solution, and then sonicated for 30 min to release the biofilms. From such tubes, dilutions of 10^−1^ up to 10^−6^ in saline were made, and then 100 μL aliquots of each dilution were seeded in TSA medium in sterile Petri dishes and incubated for 24 h. Untreated and carvacrol coated stainless steel slides were used as positive and negative control, respectively. Finally, bacteria viability was expressed as the log of CFU/cm^2^, by the following Equation (2):

CFU/cm^2^ = number of colonies × dilution × sample area (2)

For visualization of the bacterial cells attached to the sample surfaces, two samples on each treatment condition were fixed for three hours in Karnovsky solution (2.5% glutaraldehyde, 2% formaldehyde, 0.1 M sodium phosphate buffer; pH 7.2), followed by rinsing with phosphate buffer for 5 min, dehydration in a series of ethanol soaking (60% and 70% solution for 5 min and 100% for 10 min), and aseptically air dried. Afterward, the samples were coated by a thin gold-palladium film for SEM analysis, which was performed in a JEOL JSM-6010LA at an acceleration voltage of 3 kV.

## 3. Result and Discussion

### 3.1. Morphology

**Figure 2:**
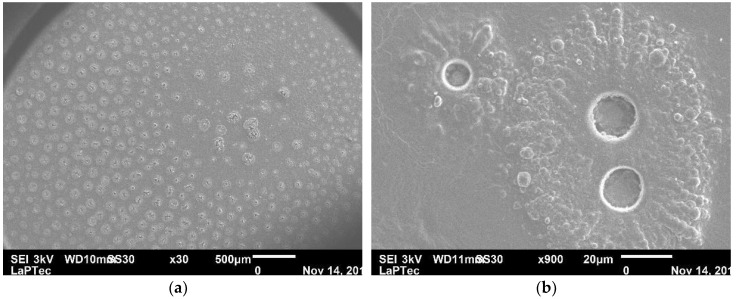
Scanning electron microscopy (SEM) micrographs of the film deposited on stainless steel slide during 45 min with (**a**) 30× and (**b**) 950× magnifications.

Figure 2 presents scanning electron micrographs of the coating deposited during 45 min, which is very similar to those obtained with samples grown under the other conditions investigated in this work (See Supplementary Materials Figures S1 and S2). As one can note, the substrate was covered by round structures uniformly distributed over the whole area. However, under higher magnification, as shown in Figure 2b, it is possible to observe that such structures are, in fact, holes surrounded by particle clusters of diverse sizes. To understand such a morphology, it is worthy to make clear that, under our experimental setup, the discharges used to deposit the coatings are filamentary [26] rather than homogeneously distributed over the whole substrate. Consequently, the holes are formed through the ablation of the coating by the intense and localized discharges. On the other hand, the clusters result from monomer polymerization driven by reactive species surrounding the filaments.

### 3.2. Roughness 

**Figure 3:**
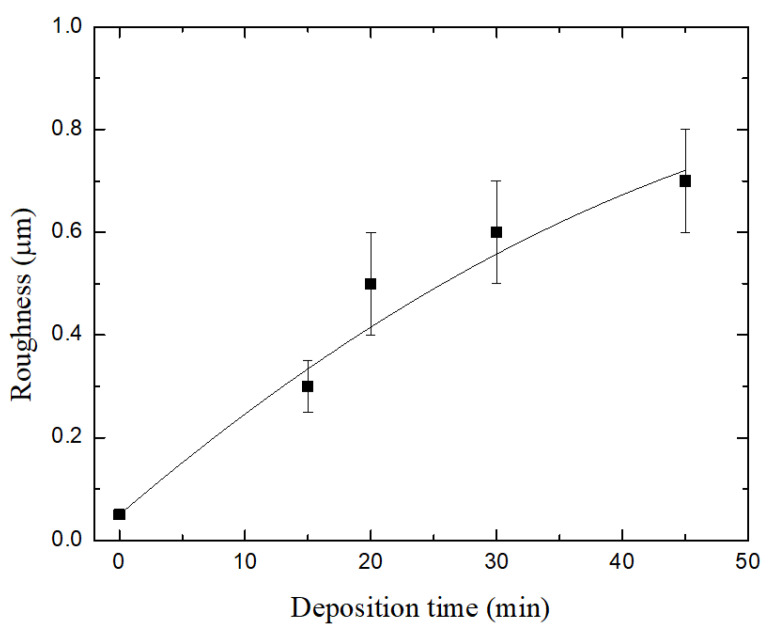
Roughness of the plasma polymerized carvacrol films as a function of deposition time. The value at *t* = 0 corresponds to the roughness of the substrate exposed to the monomer with the plasma off.

Figure 3 shows the average *Ra* roughness of the films as a function of the deposition time, *t.* As can be noted, all the films are rougher than the steel substrate exposed to the monomer with the plasma off, whose roughness is indicated by the point at *t* = 0. The observed increase of *R_a_* as the deposition time was increased is the result of the overexposure of the films to the discharge filaments for longer periods of time, causing the degradation of the surfaces, as evidenced by SEM micrographs presented in Figure 2.

### 3.3. Thickness and Deposition Rate

**Figure 4:**
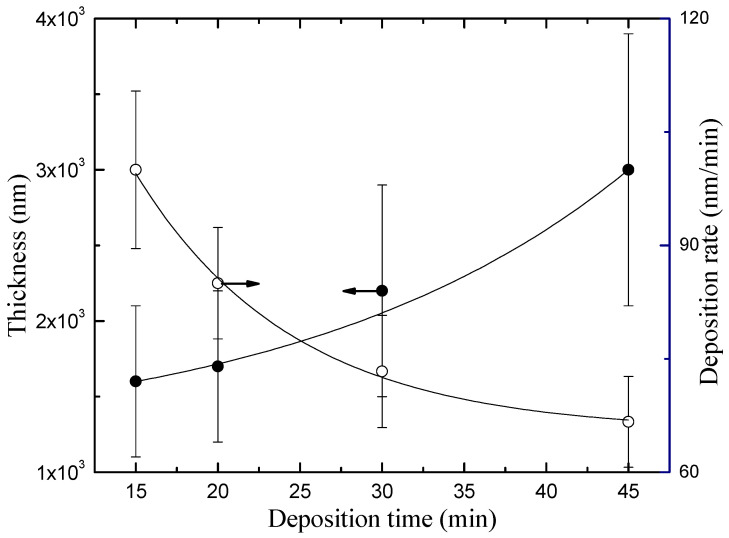
Thickness and deposition rate of plasma deposited films as a function of deposition time.

Figure 4 shows the thickness *h* of the coatings as a function of *t*. Films as thick 1.6 μm were obtained with a deposition time of 15 min. As the deposition time was increased, *h* increased, reaching nearly 3.0 μm. Previous studies [27,28] have reported that the thickness of plasma polymerized terpinen-4-ol and linalyl acetate (PLA) thin films using low-pressure radiofrequency plasmas also increases linearly with the deposition time. However, under some deposition times, the thicknesses of the coatings produced there were around one-third of the thickness obtained in our work. It is interesting to mention that, as also shown in Figure 4, the deposition rate, defined as *h/t*, decreased from 100 to 65 nm/min as the deposition time increased. In order to understand this result, it is interesting to recall that, during the deposition, the sample is exposed to large amounts of atomic oxygen and other reactive species, such as OH and O_3_, resulting from interactions between the plasma and the air surrounding the samples. Such species may etch the deposited material through chemical reactions, resulting in volatile compounds such as CO and CO_2_. In addition, the continuous bombardment of energetic species in the plasma may also cause the film densification [29]. Therefore, the results suggest that the higher the deposition time, the higher the influence of plasma on the ablation and densification of the growing material, which may result, for instance, on the holes observed in Figure 2.

### 3.4. Chemical Structure Analysis 

**Figure 5:**
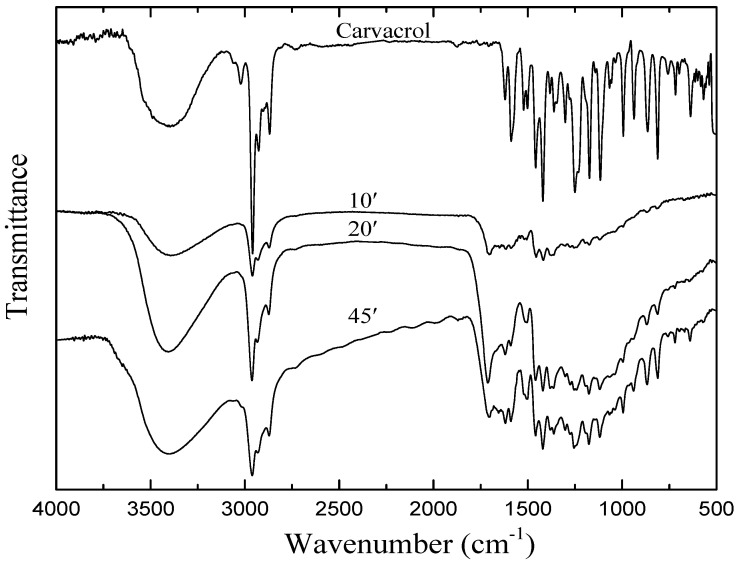
Fourier-transform infrared spectroscopy FTIR absorption spectra of liquid and plasma deposited carvacrol films grown under different deposition times.

The FTIR spectrum of liquid carvacrol used as film precursor is shown in Figure 5. On the spectrum, it is possible to note a broad band centered at 3412 cm^−1^, ascribed to O-H stretch, and strong absorptions at 1058 and 1112 cm^−1^ assigned to the presence of O-H and C-O bond stretching of phenol groups, respectively. The strong and sharp peaks in the ranges 1519–1620 cm^−1^ and 3057–3017 cm^−1^ are attributed to aromatic C=C and C-H stretching, respectively [30,31]. The peaks in the range 991–936 cm^−1^ and the peak centered at 1418 cm^−1^ indicate O-H bending vibrations overlapping with symmetric and asymmetric in-plane bending, respectively, of aromatic C-H groups. The strong band in 2959–2868 cm^−1^ indicates C-H stretching vibration of the methyl group [32,33]. In addition, the bands at 1462 cm^–1^ and 1300 cm^−1^ also appear owing to C-H asymmetric and symmetric deformation of the methyl group, respectively. On the other hand, the strong absorption at 1177 cm^−1^ indicates carbon skeletal vibration of C-(CH_3_)_2_ groups [11]. The peaks at 1363 and 1381 cm^−1^ also confirm symmetric and asymmetric deformation, respectively, of the isopropyl group.

Figure 5 also presents the spectra of films deposited under various deposition times. As one can note, the spectra of all the coatings are notably similar to that of the monomer, indicating that the main functional groups in the monomer were preserved after the deposition. The absence of the absorption at 3017 cm^−1^ and the appearance of absorption at 1705 cm^−1^ are also noticeable on the spectra of the films, which is associated with ketone functional groups. Such absorptions may result from post-plasma oxidation of free radicals trapped during the formation of the film, as well as from tautomerization [34,35].

As the deposition time increased, the intensity of almost all absorption increases, which is in agreement with the increase of the film thickness with *t*, as shown in Figure 4. Furthermore, it is interesting to point out the maintenance of hydroxyl groups on the plasma deposited films. Such radicals, which are involved on the rupture of the bacteria cell membrane, are believed to be one of those responsible for the antimicrobial activity of carvacrol. In addition, when OH containing-phenol rings penetrate the cells, they can disrupt the cytoplasmatic membrane, inhibiting the activity of several enzymes and affecting numerous mechanisms related to cell metabolism [36,37].

### 3.5. Water Contact Angle and Surface Energy

**Figure 6:**
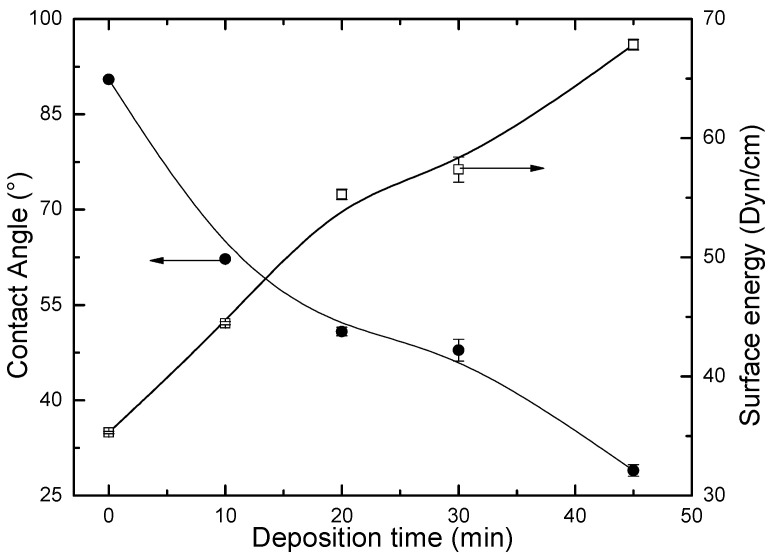
Water contact angle and surface energy of the plasma deposited carvacrol films as a function of deposition time. The values indicated at *t* = 0 correspond to results measured with the substrates exposed to the monomer with the plasma off.

The values of water contact angle and surface energy of the steel substrate exposed to the monomer with the plasma off and the films deposited with various deposition time are shown in Figure 6. As can be observed, the exposure to the plasma causes the contact angle to continuously decrease from 90.2°, measured on the surface not exposed to the plasma, to 28°, after deposition for 45 min. This increment of film hydrophilicity is the result of the enhancement of oxygen-containing groups, which leads to the rise of the surface polarity, and, consequently, to the increase of surface energy, as also shown in the figure, of films fabricated at a higher deposition time [27,38]. That is in agreement with the increase of the intensity of absorptions ascribed to hydroxyl groups with the increase of the deposition time, as shown in Figure 5. Another fact that contributes to the growth of the hydrophilicity is the increasing of film roughness [39] with *t*, as discussed in the previous sections.

As can be observed in Figure S3 of the supplementary material, the coatings are stable as the hydrophilicity conferred to the steel substrates by the deposited films is still present even 35 days after the deposition. In general, hydrophilic surfaces, as those obtained in this work, are the most suitable for many biological applications [40,41].

### 3.6. Biofilm Assays 

**Figure 7:**
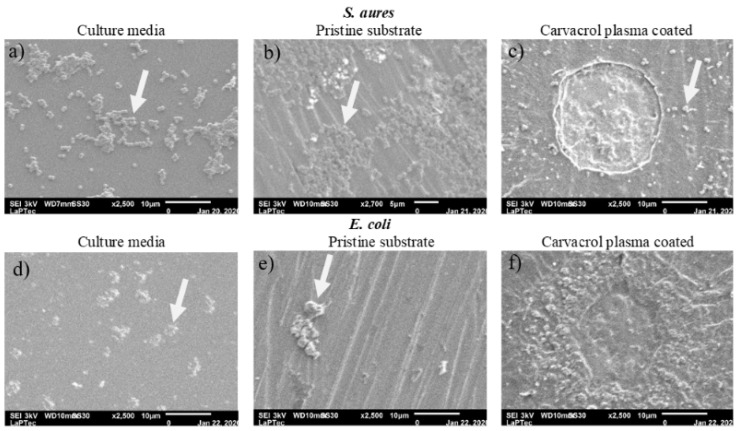
Scanning electron micrographs of colony forming units (CFUs) of *S. aureus* and *E. coli* on culture media ((**a**,**d**), respectively) as well as on stainless steel samples as-received ((**b**,**e**) for *S. aureus* and *E. coli*, respectively) and coated with plasma deposited carvacrol films ((**c**,**f**) for *S. aureus* and *E. coli*, respectively). The arrows indicate the bacterial colonies.

Figure 7 shows SEM micrographs of *S. aureus* and *E. coli* colonies after 24 h of incubation in culture media, Figure 7a,d, as well as on steel substrates as-received, Figure 7b,e, and coated with plasma deposited carvacrol films, Figure 7c,f. As can be seen, for both micro-organisms, the densities of colony forming units (CFUs) attached to pristine substrates are even higher than those observed in the culture media. In addition, it is also worth noting the formation of clusters of cells linked to each other by extracellular polymeric substances, indicating the initial stages of biofilm formation. On the other hand, in Figure 7, it is also possible to observe that the plasma deposited carvacrol coatings completely inhibited the adhesion of *E. coli* and reduced to only a few CFU of *S. aureus* on the steel surface.

**Figure 8:**
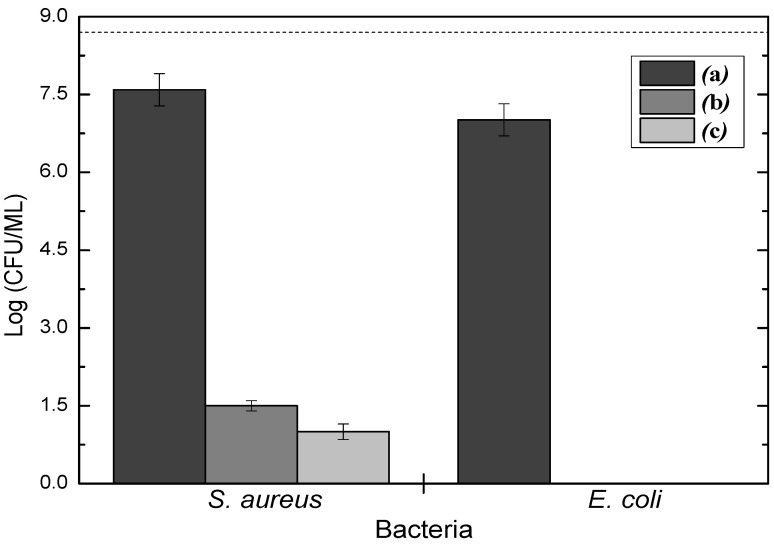
The concentration of *S. aureus* and *E. coli* on stainless-steel slides as-received (**a**), immersed for 45 min in liquid carvacrol (**b**), and coated with plasma deposited carvacrol for 45 min (**c**). The dotted line indicates the concentration of bacteria in the initial inoculum.

The effects of the coatings on the bacterial adhesion can be further evaluated with the help of Figure 8, which shows the results of quantifications of viability and proliferation of microorganisms on stainless steel slides as-received (a), immersed in liquid carvacrol for 45 min (b), and coated with a plasma deposited carvacrol film grown for 45 min (c). In this figure, the dotted line indicates the bacteria concentration in the initial inoculum. As can be noticed, all carvacrol-containing samples exhibit complete eradication of *E. coli* and reduced by about six orders of magnitude the concentration of *S. aureus*. On the other hand, the pristine substrate preserved the viability of more than 90% of both bacteria. Indeed, the biofilm eradication of the coating is similar to that obtained with the immersion of the stainless-steel slides in liquid carvacrol. However, as can be concluded from the results presented in the Supplementary Material (Figure S4), the coatings produced without plasma are totally degraded after 20 min under UV light, which is a typical procedure for material sterilization. On the other hand, no clear evidence of degradation can be observed on the plasma deposited carvacrol films even after one hour under UV irradiation. Therefore, the exposure to the plasmas results in stable coatings with bactericidal activity even after being stored in atmospheric air for more than 120 days (Figure S5).

## 4. Conclusions

The deposition of carvacrol thin film on stainless steel substrates was successfully achieved by atmospheric pressure dielectric barrier discharge plasmas. The obtained films are well-adhered to the substrate and stable under UV light and air exposure for prolonged periods. The coatings completely inhibited the formation of *E. coli* biofilms and reduced by six orders of magnitude the adhesion of *S. aureus* in comparison with the number of bacteria detected on the pristine substrate. The results of bactericidal activity, associated with the fact that carvacrol is a natural extract, make the films produced here good candidates for coatings to avoid the formation of biofilms on biomaterials and food packaging, for instance.

## Supplementary Materials

The following are available online at https://www.mdpi.com/1996-1944/13/14/3166/s1, Figure S1: Scanning electron micrographs of (a) stainless steel and of the film deposited at 30 min deposition time and 3 L/min gas flow rate (b) at 0.68 W and (c) at 0.86 W; Figure S2: EDS map of the film deposited at 0.77 W and 5 L/min gas flow rate for 45 min deposition time; Figure S3: The water contact angles, measured before and after aging for 35 days under ambient conditions, for films derived from carvacrol at 0.6 W and 5 L/min gas flow rate under different deposition times; Figure S4: FTIR spectra of (a) and (b) direct coating of carvacrol monomer, and (c) and (d) carvacrol thin film deposited at 0.54 ± 0.04 W and 4 L/min flow for 30 min deposition time on stainless steel substrate; Figure S5: FTIR spectra of plasma deposited carvacrol film deposited at 0.54 ± 0.04 W and 4 L/min gas flow rate for 10 and 45 min deposition times, measured before and after aging for 35 60 to 90 and 120 days under ambient conditions.
