# Supplementary material for: Atmospheric Pressure Plasma Chemical Vapor Deposition of Carvacrol Thin Films on Stainless Steel to Reduce the Formation of E. Coli and S. Aureus Biofilms
Source: Materials (Basel). 2020 Jul 15;13(14):3166. doi: 10.3390/ma13143166 (PMC7411687; doi:10.3390/ma13143166)
Supplement: Supplementary file 1 [file materials-13-03166-s001.pdf]

materials

MDPI

Supplementary Materials

Atmospheric Pressure Plasma Chemical Vapor Deposition of Carvacrol Thin Films on Stainless Steel to Reduce the Formation of E. Coli and S. Aureus Biofilms

Tsegaye Gashaw Getnet [1,2], Gabriela F. da Silva [3], Iolanda S. Duarte [3], Milton E. Kayama [4], Elidiane C. Rangel [1] and Nilson C. Cruz [1,*]

1 Laboratory of Technological Plasmas, São Paulo State University, Sorocaba 18087-180, SP, Brazil; tsegshchem2004@gmail.com; elidiane.rangel@unesp.br
2 Department of Chemistry, Bahir Dar University, Bahir Dar 79, Ethiopia
3 Laboratory of Environmental Microbiology, Federal University of Sao Carlos, Sorocaba 18052-780, SP, Brazil; gfiori.silva@gmail.com; iolanda.duarte@gmail.com
4 Laboratory of Plasmas and Applications, São Paulo State University, Guaratinguetá 12516-410, SP, Brazil; ekayama@gmail.com or milton.kayama@unesp.br
* Correspondence: nilson.cruz@unesp.br or nilson@sorocaba.unesp.br; Tel.: +55-15-3238-3455

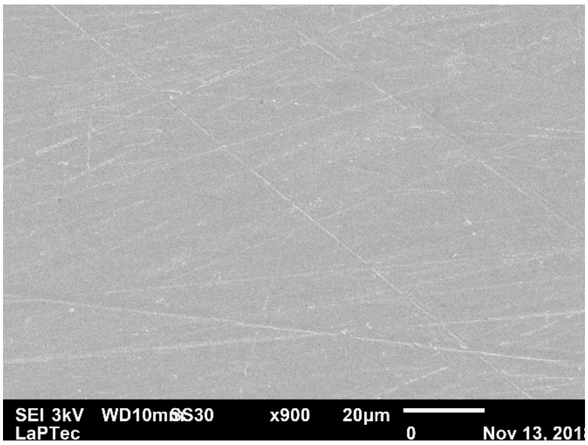

(a)

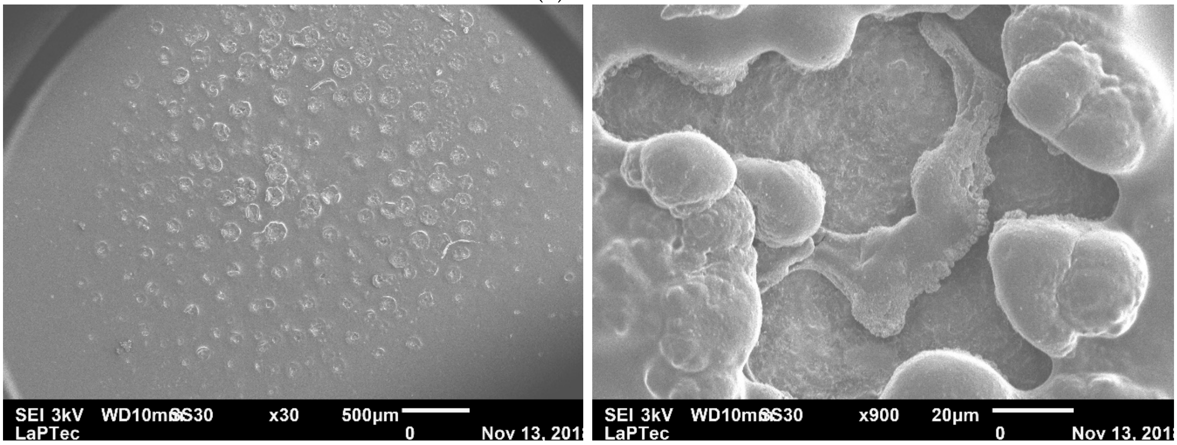

(b)

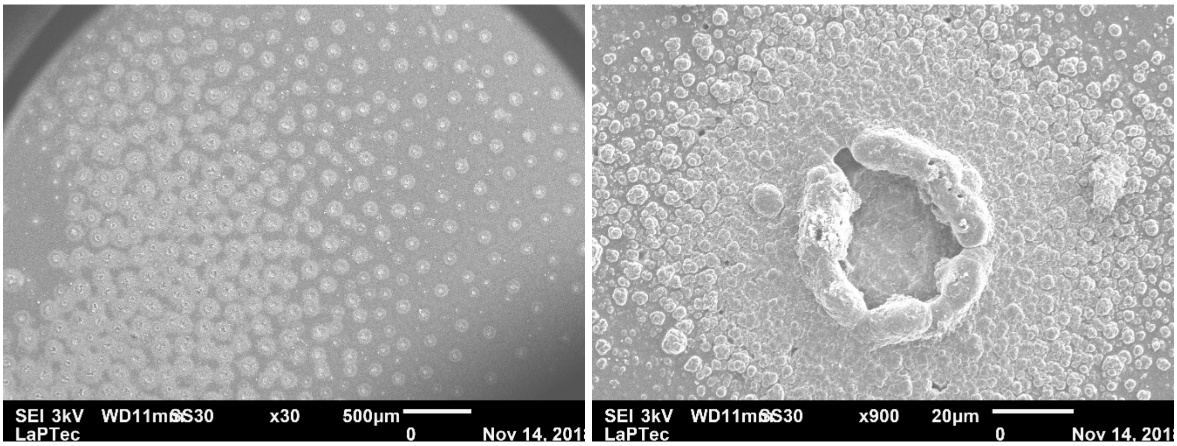

(**c**)

Figure S1. Scanning electron micrographs of (**a**) stainless steel and of the film deposited at 30 min deposition time and 3 L/min gas flow rate (**b**) at 0.68 W and (**c**) at 0.86 W.

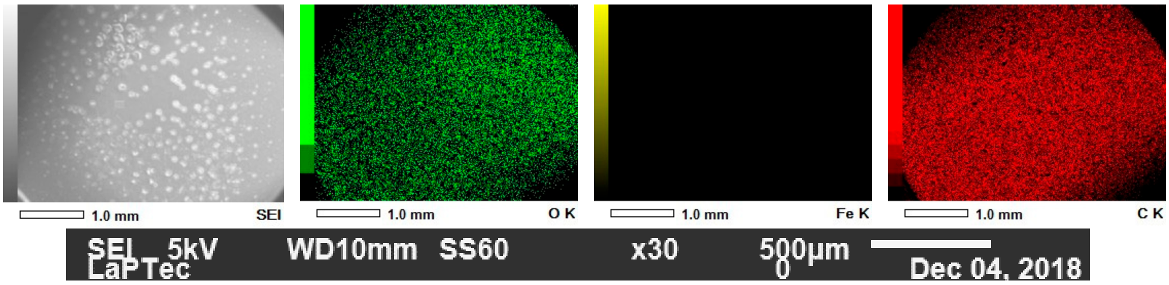

Figure S2. EDS map of the film deposited at 0.77 W and 5 L/min gas flow rate for 45 min deposition time. The EDS mapping is operated at 5 kV.

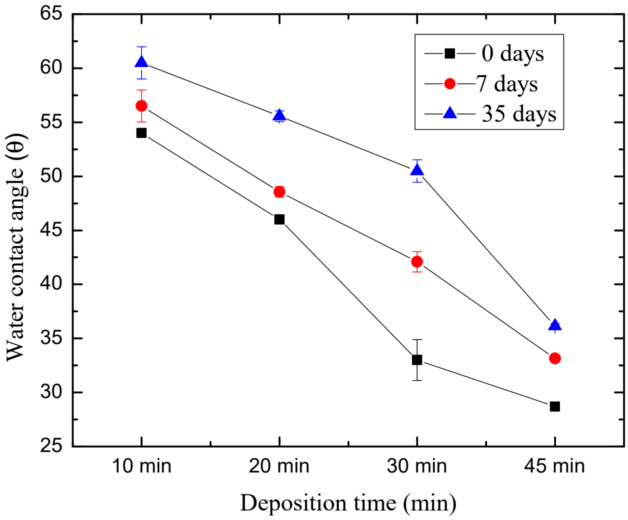

Figure S3. The water contact angles, measured before and after aging for 7 and 35 days under ambient conditions, for films derived from carvacrol at 0.6 W and 5 L/min gas flow rate under different deposition times. The error bars result from measurements performed on three series of samples.

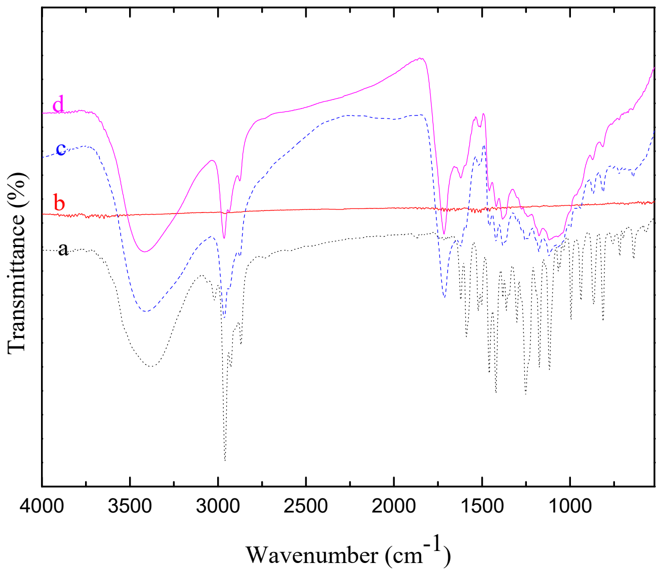

Figure S4. FTIR spectra of (a and b) direct coating of carvacrol monomer, and (c and d) carvacrol thin film deposited at 0.54 ± 0.04 W and 4 L/min flow for 30 min deposition time on the stainless steel substrate. The dashed and solid lines are before and after exposing the film and monomer to high thermal UV-lump at 65 °C, respectively.

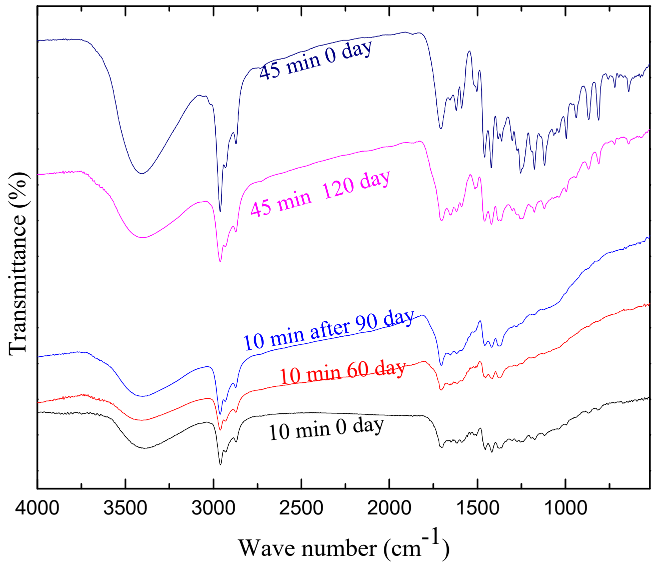

Figure S5. FTIR spectra of polycarvacrol film deposited at 0.54 ± 0.04 W and 4 L/min gas flow rate for 10 and 45 min deposition times, measured before and after aging for 60 to 90 and 120 days under ambient conditions.

© 2020 by the authors. Submitted for possible open access publication under the terms and conditions of the Creative Commons Attribution (CC BY) license (http://creativecommons.org/licenses/by/4.0/).
